# Exploring the potential for gene therapy in Cav1.4-related retinal channelopathies

**DOI:** 10.1080/19336950.2025.2480089

**Published:** 2025-03-25

**Authors:** Matthias Ganglberger, Alexandra Koschak

**Affiliations:** Pharmacology and Toxicology, Institute of Pharmacy, University of Innsbruck, Innsbruck, Austria

**Keywords:** Voltage-gated ion channel, L-type calcium channel, photoreceptor, ribbon synapse, gene therapy

## Abstract

The visual process begins with photon detection in photoreceptor outer segments within the retina, which processes light signals before transmission to the thalamus and visual cortex. Cav1.4 L-type calcium channels play a crucial role in this process, and dysfunction of these channels due to pathogenic variants in corresponding genes leads to specific manifestations in visual impairments. This review explores the journey from basic research on Cav1.4 L-type calcium channel complexes in retinal physiology and pathophysiology to their potential as gene therapy targets. Moreover, we provide a concise overview of key findings from studies using different animal models to investigate retinal diseases. It will critically examine the constraints these models present when attempting to elucidate retinal channelopathies. Additionally, the paper will explore potential strategies for addressing Cav1.4 channel dysfunction and discuss the current challenges facing gene therapy approaches in this area of research.

## Introduction

The retina, a complex neural network lining the back of the eye, serves as the crucial first stage in visual processing, transforming light signals into electrical impulses that the brain can interpret. This sophisticated tissue contains specialized photoreceptor cells – rods and cones – that detect light and initiate the visual pathway, along with other neural cells that perform initial processing of visual information. Given its critical role in vision and its unique characteristics, during the last decades, the retina has become a prime target for innovative gene therapy approaches and has made significant progress, exemplified by the FDA approval of voretigeneneparvovecrzyl (Luxturna) in 2017 for treating RPE65-related retinal dystrophy [1–3] for review see [4,5]. This pioneering achievement is likely to inspire researchers to explore novel gene therapy targets within the retina. Specifically, it may encourage investigations into retinal ion channels, with a focus on voltage-gated calcium channel (VGCC) genes. Examples include *CACNA1F* (OMIM:300110), which encodes the Cav1.4 voltage-gated L-type calcium channels (LTCC), as well as their auxiliary Cavβ2 and α2δ-4 subunits, encoded by the *CACNB2* (OMIM:600003) and *CACNA2D4* (OMIM:608171) genes respectively.

Cav1.4 LTCCs play a crucial role in retinal neurotransmission at photoreceptor terminals [6,7] for review see [8]. Their functional properties are regulated by various factors, including accessory subunits or alternative splicing [9]. Of note, pathogenic variants of the *CACNA1F* gene are associated with X-linked retinal disorders such as congenital stationary night blindness type 2 (CSNB2, OMIM: 300071) [9–12] and can lead to a wide range of channel dysfunction phenotypes: from typical loss-of-function attributed to a reduced number of functional channels localized at the plasma membrane to an apparent gain-of-function i.e. due to a hyperpolarizing shift of the IV-curve and increased single-channel activity [13].

CSNB2 patients show a reduced but still recordable dark-adapted rod ERG, along with an electronegative dark-adapted rod-cone ERG. Their light-adapted cone ERG is also diminished. Patients often report impaired dim and night vision, but not all of them explicitly mention night blindness. High myopia is common, and visual acuity is generally decreased. Importantly, both rod- and cone-related visual functions are affected. Some patients may additionally experience nystagmus, strabismus, or photophobia [10]. It is worth noting that the severity of these symptoms can vary considerably among individuals with *CACNA1F* mutations, reflecting the complex nature of this condition. The subtle variations in clinical presentation may be explained by differences in the underlying functional defect. Therefore, these differences must be considered when developing gene therapy approaches aimed at restoring channel function.

Cav1.4 channels present a compelling target for treating retinal disorders due to several key factors. Their predominant localization in retinal neurons offers a high degree of specificity, allowing for targeted interventions. These channels play a critical role in both calcium signaling and synaptic organization, making them essential for proper synaptic transmission in the retina. The direct link between pathogenic variants in the *CACNA1F* gene and retinal disorders provides a clear genetic basis for therapeutic approaches. Addressing Cav1.4 dysfunction has the potential for broad impact, as it could ameliorate multiple aspects of retinal disorders, including both synaptic structure and function. Furthermore, developing treatments that target Cav1.4 channels could not only alleviate disease symptoms but also enhance our understanding of the channel’s physiological role in retinal function. Such multifaceted approach underscores the significant therapeutic potential of focusing on Cav1.4 channels in the treatment of retinal disorders.

### Role of voltage-gated calcium channels in the transmission at the photoreceptor synapse

**Figure 1.**
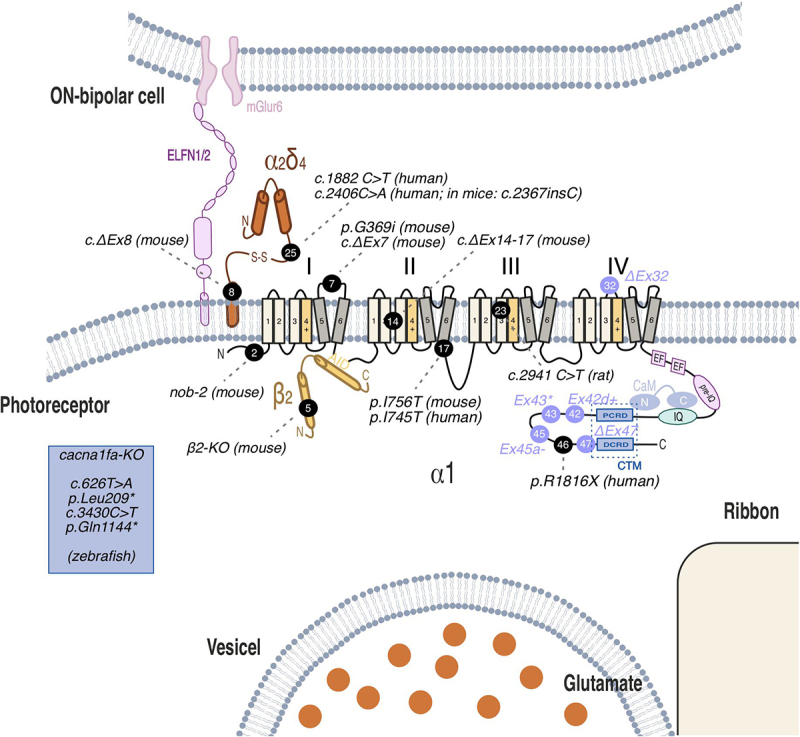
Protein topology of Cav1.4 illustrating the different variants and their respective exons discussed in this review. Alternatively spliced exons are highlighted in blue. The scheme also includes the intracellular β2 subunit, the extracellular α2δ-4 subunit, and the ELFN protein. Potential interaction site with calmodulin (CaM) and the IQ domain is indicated.

The retina, a complex neural network lining the back of the eye, serves as the crucial first stage in visual processing, transforming light signals into electrical impulses that the brain can interpret. This sophisticated tissue contains specialized photoreceptor cells – rods and cones – that detect light and initiate the visual pathway, along with other neural cells that perform initial processing of visual information. Given its critical role in vision and its unique characteristics,

Transmission at ribbon synapses is initiated by variations in membrane potential and is dependent on Cav1 LTCCs. This differs from the Cav2 channels found in conventional synapses, which are primarily responsible for rapid, phasic neurotransmitter release in response to action potentials [8,14,15]. At synaptic junctions, Cav1 channels form clusters positioned strategically beneath the synaptic ribbon [14,16]. Such precise arrangement facilitates the influx of Ca^2+^ ions, initiating vesicle fusion with the plasma membrane. Consequently, glutamate is released into the synaptic cleft. Research indicates that the mobility of these channels can significantly affect neurotransmitter release, suggesting that regulating their movement may enhance synaptic efficacy [15]. Notably, Cav1 channels at photoreceptor ribbon synapses possess specialized characteristics tailored to efficiently transmit analog signals, like light intensity, distinguishing them from Cav1 channels in other cell types. These distinctive properties arise from specific domains within the Cav1 protein and its interactions with accessory proteins comprising the channel complex (Figure 1, Cavβ2 and α2δ-4 [17–19]).

Several studies using immunohistochemistry and in situ hybridization have indicated the presence of all Cav1 subunits at mouse retinal ribbon synapses [6,20–24] for review see [25]). This has initially led to a debate about the specific identity of the Cav1 calcium channels in this location. However, studies strongly indicate that Cav1.4 is the primary calcium channel responsible for neurotransmitter release at these specialized synaptic structures in the mouse retina [8,9]. Recently, Grabner and colleagues, using 3D minfluxnanoscopy, also reported that Cav1.4 channels are arranged bilaterally, forming two parallel rows spaced 116 nm apart; a distance sufficient to prime and release synaptic vesicles at both sites at the base of the synaptic ribbon [26].

Maddox and colleagues [27] highlighted the structural significance of Cav1.4 channels at the ribbon synapse, beyond their role in signal transmission. A more recent study from their research group also explored a compensatory mechanism in cone ribbon synapses by introducing a non-conducting Cav1.4 channel (G369i [28]). By assessing the transsynaptic response in horizontal cells, the study found no light response in horizontal cells within the Cav1.4-KO retina, while On and Off responses were detected in the G369i mutant. Their data suggested the involvement of other channels in compensating for cone ribbon synapse transmission, because the biophysical characteristics resembled T-type channels rather than Cav1.4 ICa. Patch-clamp experiments in Cav1.4-G369i mice using a step depolarization protocol revealed a calcium current (ICa) with faster inactivation and a hyperpolarizing shift of about 10 mV compared to wild-type cones. However, such currents were not observed in wild-type mice or other mammals like macaques or ground squirrels, suggesting that T-type channels have a relatively small effect and may not be detectable in the presence of the Cav1.4 [28]. In contrast, Davidson et al. [29] provided functional evidence for T-type channels in cone photoreceptors through patch-clamp recordings. Using a ramp voltage protocol from −89 mV (800 ms) to +49 mV, they observed two peaks in ICa recordings comprising a low (LVA) and high voltage activated (HVA) ICa component. The contribution of T-type channels was further confirmed by specific blockers, like nickel and Z944, which reduced the ICa current. Additionally, the physiological properties of the pharmacologically isolated ILVA were consistent with heterologous expression studies [30,31]. Furthermore, several studies have identified the presence of Cav3.2 mRNA (*cacna1h*) in mouse cones, while no T-type channels were detected in rods [28,29,32]. However, the group of Norbert Babai reported that the low-voltage activated (LVA) current component they measured began activating at approximately −50 mV, with peak currents around −40 mV, whereas the high-voltage activated (HVA) current component peaked at approximately −20 mV. They suggested that depolarization at light offset first activates T-type channels, which in turn activates HVA LTCCs. T-type calcium channels might therefore shift the ICa activation to more negative potentials and extend the light-responsive membrane potential range of cone photoreceptors.

### Functional properties of Cav1.4 L-type calcium channel complexes

A small number of VGCCs that activate rapidly at moderately negative voltages and exhibit slow inactivation are sufficient to enable tonic glutamate release as previously noted in invertebrate retinas [30,33,34]. In line with these reports, heterologous expression studies in different systems have demonstrated that Cav1.4 currents activate around −40 mV with rapid activation kinetics [31,35,36], also under physiological temperatures [37]. As a fundamental autoregulatory mechanism, Ca^2+^ ions within cells play a crucial role in modulating the activity of VGCCs through calcium-dependent inactivation (CDI). This regulatory process is mediated by calmodulin, a widely distributed protein that binds calcium, which binds to the IQ motif located in the calcium channel’s C-terminus [38–45]; for review see [46,47]. Of note, in Cav1.4 channels CDI is absent [48,49] while the voltage-dependent inactivation (VDI) of Cav1.4 is slow [31,35,36]. The lack of CDI in Cav1.4 channels is due to a specific inhibitory domain located in their C-terminus. This domain, referred to as either the inhibitor of CDI (ICDI [49]) or C-terminal modulation (CTM [50]) domain, actively suppresses CDI and affects other gating properties [13,49,50]. This mechanism was first described by Hulme and colleagues who observed a similar interacion in Cav1.1 and Cav1.2 [51,52]. The modulation occurs through an interaction between proximal and distal C-terminal regulatory domains whereby the distal C-terminus competes with apoCaM for binding near the channel’s IQ domain, inhibiting CDI by displacing CaM from its interaction with the channels [53]; for review see [46]. CTM in Cav1 calcium channels plays a significant role beyond just regulating CDI. It exerts a multifaceted influence on channel function, also affecting activation gating and open probability [13,48,50–52,54,55]. Studies on pathogenic Cav1.4 variants lacking the CTM have provided valuable insights into these regulatory functions. For instance, analysis of the R1827X variant, which is truncated and lacks the CTM, revealed significant changes in channel behavior [13]. Single-channel recordings demonstrated that while open times were unaffected, there was a notable increase in open probability for R1827X compared to wild-type channels at any given test potential. This observation aligns with whole-cell recordings showing the expected leftward shift in the current–voltage curve for R1827X [13]. Of note, similar effects of the CTM have been observed also in Cav1.3 channels [48,54] where alternatively spliced short variants results in pronunced CDI and channel activation at more negative potentials [48,54]. The latter is due to an increase in the open probability, as in Cav1.4 [54]. There is also evidence that Cav1.4 channels show reduced frequency-dependent inactivation when the CTM is intact [13]. Together these properties are essential for the specialized function of Cav1.4 channels in sensory systems, particularly in photoreceptors, where sustained calcium influx is required for proper signal transmission.

Alternative splicing is a crucial mechanism that enhances the functional diversity of VGCCs, allowing them to adapt to various physiological requirements across different tissues and cell types [56,57]. This process is particularly prevalent in Cav1.2 and Cav1.3 channels, which are widely shown in multiple tissues and might also correlate with such broad tissue distribution, enabling fine-tuning of channel properties to meet specific cellular needs (for review see [25,58]). Cav1.4 (and also Cav1.1) channels have more restricted localization patterns, primarily in skeletal muscle and retina, respectively. This narrower distribution might suggest a reduced necessity for diverse channel variants [25]. Despite its restricted localization pattern, Cav1.4 still demonstrates multiple-splice variants. Some of these variants have undergone functional characterization [17,59]. Alternative splicing in Cav1.4 channels can have a wide range of functional consequences, from subtle alterations to major changes in channel properties. Minor modifications result in slight changes in activation or inactivation kinetics e.g. in C-terminal variants like Ex45a- and Ex42d+ (Figure 1) which have minor sequence alterations and show little to no effect on current–voltage relationships or CDI properties [17,59]. Variants with substantial deletions in the C-terminus (Ex43* and ΔEx47) (Figure 1) show pronounced CDI and a hyperpolarizing shift in voltage-dependent activation as expected [60]. Voltage sensor modifications like in the ΔEx32 variant, affecting the IVS3-IVS4 linker (Figure 1), exhibit a substantial hyperpolarizing shift in the current–voltage curve (so far only tested in a chimeric approach [61]).

The potential interplay between pathogenic and splice variants is crucial because it can help in elucidating how genetic mutations might lead to diseases by affecting the normal function of cellular channels through aberrant splicing. This knowledge is also important for developing targeted therapies or other interventions and has been largely understudied particularly in terms of how splice variants affect the function of channels harboring pathogenic variants. A notable report investigating this interplay examined splice variant-dependent effects on one CSNB2 variant referred to as Cav1.4-IT in the following ([62]; for details see also below). The researchers compared the mutation’s impact on the canonical full-length Cav1.4 with a variant lacking exon 47 (ΔEx47). Their findings revealed a hyperpolarizing shift in half maximal voltage of activation for both, full-length (−25 mV) and ΔEx47 (−16 mV) channels carrying the Cav1.4-IT variant. The most intriguing aspect of these findings is the cumulative nature of the change in activation, leading to a very negative activation threshold for Cav1.4-IT-ΔEx47 (−36 mV). These findings underscore the importance of considering both pathogenic and splice variants when assessing channel function and associated pathologies. Future research should therefore focus on i. investigating a wider range of mutations and splice variants across different ion channels ii. Examining, how these interactions might contribute to disease severity or progression iii. developing more comprehensive models to predict the combined effects of mutations and splice variants on channel function. Understanding these complex interactions could lead to more accurate novel therapeutic approaches for channelopathies and related disorders.

Cav1.4 channels form a complex with β2 and α2δ4 accessory subunits as indicated by multiple lines of evidence e. g. α2δ4 has been shown to co-localize with Cav1.4α1 and β2 subunits in structures resembling photoreceptor synaptic ribbons [63] and expression profiling confirmed that Cav1.4, β2, and α2δ-4 were by far the most abundant isoforms in wild type and mutant retinas [64]. Ablinger and colleagues [65] investigated the pattern of the α2δ-4 subunit in cultured neurons, concluding that this accessory subunit is localized at presynaptic synapses. Interestingly, overexpression of α2δ-4 did not increase the presynaptic clustering of endogenous Cav2.1 channels, again highlighting its predominant interaction with Cav1.4. However, α2δ-4 proteins were capable of rescuing glutamatergic synapse formation and function in α2δ -KO neurons. Notably, α2δ-4 is not exclusively found in retinal tissue and the pineal gland; low levels of cacna2d4 mRNA were also detected in the hippocampus, with expression levels upregulated during development [66]. Functional studies have shown that Cav1.4 mediated currents were increased when α2δ4 or also α2δ1 subunits were co-expressed [17,18]. Lee and colleagues have further reported that this similarity between α2δ4- and α2δ1-mediated effects persists independent of the β2 subunit isoform co-expressed [17]. Of note, a spontaneous frameshift mutation in mice (c.2367insC) causing a truncation of the α2δ4 protein resulted in low protein levels, explaining the synaptic disorganization and defective retinal signal transmission in these animals [67,68]. This is caused by an alternative form of exon 25, known as exon 25b, that produces a variant of the α2δ4 protein that lacks the δ peptide. This δ peptide is essential for anchoring the protein to cell membranes. Additionally, without the δ peptide, the remaining part of the protein is likely to be secreted. In mouse retinas carrying the c.2367insC mutation, the proportion of transcripts including E25b is increased (termed c.2451insC in [18]). This variant does not significantly enhance ICa contrary to the full-length α2δ4 protein [18]). In humans, mutations in exon 19 and 25 of the *CACNA2D4* gene, which encodes α2δ-4 subunits, have been linked to retinal dystrophy [67].

In addition to the auxiliary subunit α2δ-4, cytosolic β subunits are crucial modulators of VGCC properties. They increase the open probability, hyperpolarize the voltage-dependence of activation [69,70] and enhance the number of channels on the plasma membrane through various mechanisms [65,66,71] leading to a several-fold increase in macroscopic ICa; for a review see [72]. All β subunits also promote VDI, with the exception of the splice variants β2a and β2e, which have the opposite effect by slowing down and reducing VDI [73–75]. In the retina, several β2 splice variants have been identified, including β2×13, which causes stronger VDI of Cav1.4 currents compared to β2a albeit both are associated with the membrane due to palmitoylation of the N-terminus [76]. More recently, Seitter and colleagues identified another β subunit splice variant in the retina, termed β2i, which is the most abundant in the retina and structurally similar to β2×13, but with a differently spliced N-terminus (Exon2E). The amino acid sequence of this N-terminus suggests membrane interaction due to the high number of hydrophobic residues. Unlike β2a, which possesses cysteine residues allowing for palmitoylation [77], and β2e, which has numerous positively charged amino acids that promote membrane association [78], the β2i subunit lacks these features. However, VDI was comparable in all membrane-associated β splice variants. Notably, among all splice variants, β2i displays a distinct clustering pattern in a heterologous expression system [19]. This clustering might explain its exclusive localization in the retina at the active zone of the ribbon synapse.

### Cav1.4 channels and their auxiliary subunits in photoreceptor synapse development

Cav1.4 channels play a critical role in the development and fate of retinal photoreceptors. The proper function and localization of these channels is essential for photoreceptor maturation. Various studies using different Cav1.4 mutant animal models have demonstrated that in the absence of functional Cav1.4 channels, photoreceptor ribbon synapses largely remain in an immature state [10,79,80]. These studies have employed various forms of Cav1.4 variants (Figure 1, Table 1): Cav1.4-deficient mice (Cav1.4 KO [64,81–85]) mice with a naturally occurring mutation leading to significantly reduced Cav1.4 protein levels (nob-2; no b-wave 2 [86]), mice carrying a gain-of-function point mutation (Cav1.4-IT (mimicking the human I756T variant (see below) [64,85,87–89])) or a non-conducting Cav1.4 mutation (Cav1.4-G369i [27]). Research on Cav1.4 has expanded to include multiple species beyond mice. In rats, a naturally occurring mutation (c.2941 C>T; p.R981X [79]) was studied. These mutant rats lack a scotopic b-wave but retain cone electroretinograms (ERG) with reduced amplitude. Morphologically, affected rats fail to develop dendrites in second-order neurons, a feature not observed in mice [79,90]. Zebrafish have also contributed to our understanding of Cav1.4-related blindness. Mutations in the *cacna1fa* (wud) gene, a paralog of human *CACNA1F*, cause molecular defects associated with vision loss. These mutations result in abnormal ERGs specifically in cone cells [80]. Together, also this multi-species approach has provided valuable insights into Cav1.4’s role in visual function.

**Table 1.** Summary of phenotypes from previously published mouse models used to study the effects of the Cav channel complex in the mouse retina.

Gene	Mouse model	Phenotype	Reference
CACNA1F	Cav1.4-Knock out	Reduced Cav1.4 protein levels	[64,81–85,101]
		Lacking invaginating postsynaptic neurons, Floating ribbons	
	(Cacna1fΔEx14–17)	ERG: reduced a-wave; missing b-wave	
	(Cacna1fΔEx7)	Reduced responsiveness of ganglion cells	
		Absence of visually evoked activation in the cortex	
		HC and BC neurite sprouting, only immature (punctate) ribbons	
	(nob2)	ERG: detectable but reduced b-wave and oscillatory potentials; a-wave normal	[86,102]
		Normal optokinetic response	
		B-wave under dark adapted conditions is similar to that seen in CSNB2 patients with pathogenic CACNA1F variants	
	Cav1.4-Knock in	ERG: reduced b-wave amplitude at the highest light intensity.	[27,28]
	(Cav1.4-G369i)	Vision-guided behavior: normal at photopic conditions, but not scotopic. Increased protein appearance: mGluR6, TRPM1; Cav1.4 and bassoon.	
		HC and BC neurite sprouting	
		Larger cone synapses; shorter and floating ribbons in rods and larger ribbons in cones; more vesicles in cone ribbons	
	(Cav1.4-I756T)	ERG: reduced a wave, reduced b-wave at photopic and scotopic responses	[64,85,87–89]
		Altered ganglion cell response, HC response from cone synapses: normal	
		Reduction in expression of Cacna1f, β2 and α2δ-4	
		Thinner ONL → progressive retinal degeneration,	
		Shorter cones, HC and BC neurite sprouting, develop ectopic neurites	
		HC maintained their synaptic contacts with cones.	
		P13: Ribbon structure similar to WT, P15: many ribbons immature, but some mature, floating ribbons in cones.	
		ERG similar in human patients, however retinal degeneration was not shown in humans.	
CACNB2	β2-Knock out	ERG: B-wave amplitude reduced, but retain some level of signal transmission in rods; a-wave remained unaffected.	[95,96]
		Reduced Cav1.4 protein levels	
		Thinner OPL and dot-like ribbon synapse proteins (e.g. Cav1.4, ribeye, and β-dystroglycan)	
		NF200 staining indicated HC axon sprouting	
		Ultrastructural analysis of rods showed a loss of ribbon-shaped structures, no invaginations of second-order neurons; However, cone synapses were largely spared; immature ribbons in rod photoreceptors.	
CACNA2D4	α2δ-4-Knock in	ERG: Reduction in the scotopic a- and b-wave	[18,68]
	(c.2367insC/c.2451insC)	Decreased α2δ-4 protein levels; truncation of the α2δ-4 protein; Increased the proportion of Ex25b transcripts	
	α2δ-4-Knock out	ERGs: no photopic b-wave, no scotopic b-wave	[92]
		Diminished presynaptic Ca^2+^ signals in rod spherules	
		Longer time to detect the escape platform in a visually guided swim assay under photopic conditions.	
		Decreased α2δ-4, PSD-95 protein levels	
		Gradual loss of presynaptic calcium channels in both rod and cone photoreceptors.	
		Cav1.4 labeling punctate at P10 and P14, but present in the OPL during early developmental stages. However, by P21, the Cav1.4 labeling was indistinguishable to WT; Normally distributed ribbons	
		Cones: α2δ-4 essential for Ca1.4 channel function, but not required for ribbon organization or synaptic transmission.	
		EM: Most cone ribbons lacked a synaptic triad and that cone bipolar cells were sprouting.	
	α2δ-4-Knock out	ERG: normal a- wave; no scotopic b-wave, reduced photopic b-wave	[91,110]
	(α2δ-4ΔEx8)	Longer time to detect the escape platform in a visually guided swim assay under photopic conditions.	
		Reduced Ca^2+^ signals in rod photoreceptors	
		No light-evoked responses in ON RBC and ON-CBC but in OFF-CBC	
		Reduced protein levels (e.g. Cav1.4, CTBP2, ELFN1, mGlur6, TRPM1)	
		BC neurite sprouting	
		Ribbons in cone pedicles of α2δ-4-KO mice appear mostly normal in structure.	
		Ribbon structures in cones appeared unaffected.	
		Less than one-third of cone ribbons show the expected triadic organization of processes at ribbon sites. Reduced photopic b-wave.	
		In patients, α2δ-4 variants cause cone- rod dystrophy	
ELFN	ELFN1-KO	ERG: Loss of scotopic transmission, while photopic transmission remains unaffected.	[91,110]
	ELFN2-KO	Did not disrupt transmission from cones to ON-cone bipolar cells.	
		Exclusively localized in cone photoreceptors.	
		However, a double knockout of both ELFN1 and ELFN2 completely prevented synaptic transmission in cones.	

The development of ribbon synapses in photoreceptors involves a complex process that is influenced by the function of Cav1.4 calcium channels. Studies have provided insights into the role of these channels at different stages of ribbon synapse formation and maturation. In the early stages of development, the initial formation of ribbon structures appears to be largely independent of Cav1.4 channel activity. This is evidenced by the normal development of ribbon precursors in both wild type and Cav1.4-KO mice during this period [85]. However, Cav1.4-KO mice exhibit notable changes in ribbon structure after postnatal day (P)8. While wild-type mice display characteristic elongated ribbons anchored to the membrane, Cav1.4-KO mice show only circular ribbons. At no point during development ribbons were found to be associated with GluR2-labeling on bipolar cells, indicating a significant disruption in synaptic organization [85]. Similar synaptic alterations were observed in Cav1.4-IT retinas. The ribbon structure in WT and Cav1.4-IT mice remains comparable up to P13, but it diverges at P15. At this stage many Cav1.4-IT ribbons displayed an immature morphology [85]. This developmental difference highlights a critical period for ribbon synapse maturation that depends on the appropriate activation properties of Cav1.4 channels. As indicated these findings have been consistently reported across multiple studies conducted by various research groups, highlighting the significance of Cav1.4 channels in retinal photoreceptor development and function.

A study using Cav1.4-G369i mice, which express a non-conducting variant, has provided further insights into the role of Cav1.4 [27]. In Cav1.4-G369i mice, Cav1.4 channels were properly targeted to photoreceptor synapses and, despite the absence of current, adult rod and cone ribbons still form in these mice. However, in rods, ribbons remain shorter in size, contrary to cone ribbons, which were enlarged. Additionally, more vesicles were associated with cone ribbons due to a larger cone synapse size [27,28]. These observations indicate that certain aspects of ribbon synapse development may not depend on the calcium-conducting function of Cav1.4 channels, but rather on their physical presence in the structure. This suggests that Cav1.4 channels might play a dual role in synapse development: one that relies on their ion-conducting properties, and another that is based on their structural contribution to the synaptic architecture. The latter function appears to be sufficient to support some developmental processes in ribbon synapses, even when the channel’s primary calcium-conducting role is compromised. This refined view of Cav1.4’s role offers key insights into retinal ribbon synapse formation with implications for understanding and potentially treating related visual disorders.

The critical role of α2δ-4 subunits as essential auxiliary components of Cav1.4 L-type calcium channels (LTCCs) in rod photoreceptor synaptogenesis is reinforced by experimental evidence [91,92]. Studies have shown that the deletion of either α2δ-4 or Cav1.4 leads to remarkably similar alterations in the structure of rod synaptic ribbons. This parallel effect strongly indicates that both proteins are not only crucial but potentially interdependent in their function of maintaining the proper organization and development of these specialized synapses in rod photoreceptors.

Pathogenic variants in the *CACNA2D4* gene, which encodes the α2δ-4 subunit, researchers observed cone – rod dysfunction in mice and retinal cone dystrophy in humans [67,68,93]. Kerov and colleagues showed that α2δ-4 plays a crucial role in clustering presynaptic Cav1.4 channels at photoreceptor ribbons. However, α2δ-4 is not necessary for the forward trafficking of Cav1.4 to the photoreceptor [92]. The lack of Cav1.4 channels, along with incorrect localization of presynaptic and postsynaptic proteins, appears to be a crucial factor in the significant alteration of ERG responses observed in α2δ-4 knockout mice. Notably, these mice exhibited no detectable b-waves in their ERG readings, highlighting the severe functional consequences of α2δ-4 deficiency on retinal signaling pathways [92]. Another finding from Wang et al. [91] demonstrated that targeted deletion of exon 8 in the α2δ-4 gene (α2δ-4-KO) resulted in a selective disruption of signal transmission in rod photoreceptors while sparing cone function. This genetic manipulation prevented the interaction between α2δ-4 and ELFN1, a protein known to be essential for the specific synaptic wiring of rod photoreceptors into the retinal circuitry [94] (see also below).

Research conducted on retinas from β2 global knockout mice (β2-KO; with cardiac-specific expression of the β2 subunit under the αMyHC promoter [95,96]) revealed changes typical of CSNB2, including altered ERG readings, a thinner outer plexiform layer (OPL), and immature ribbon synapse proteins. (e.g. Cav1.4, ribeye, and β-dystroglycan) (see above). Ultrastructural analysis of rods showed a loss of ribbon-shaped structures in the majority of the presynaptic terminals. Additionally, the invaginated postsynaptic dendritic complex was absent. In contrast, cones retained their characteristic arc-shaped ribbon structure but exhibited changes in PNA localization, suggesting only minor alterations in cone photoreceptors. These results underscore the critical importance of the β2 subunit in the development of ribbon-type synapses, especially in rods [96].

Recent studies have expanded our understanding of β2 subunits beyond their role in ribbon synapses. They are also found in retinal epithelium cells [97], ARPE19 cells and human stem cell-derived mature retinal epithelium cells [98]. Two mutations in the *CACNB2* gene have been linked to proliferative diabetic retinopathy. Research on β2-KO models showed decreased β2 mRNA levels and reduced VEGF secretion in ARPE19 cells [99]. Further studies using L-type channel blockers and activators demonstrated a relationship between calcium channel activity and VEGF secretion [100]. These findings suggest that the β2 subunit may play a role in calcium channel-mediated regulation of VEGF secretion in retinal epithelial cells.

### Cav1.4-related mouse models for studying CSNB2 and their effectiveness in reflecting the human disease

Our knowledge of CSNB2 pathology has been significantly advanced through the study of various mouse models. Two key Cav1.4-KO models have been particularly instrumental in this research (see also above [81,83]); however, it is important to note that the phenotype observed in these mice is considerably more severe than what is typically seen in human patients with CSNB2. The Cav1.4-KO mice exhibit several distinctive features, as such functional blindness, significantly reduced responsiveness of ganglion cells to normal light stimuli and absence of visually evoked activation in the cortex [81,83,101]. These findings, although extreme in comparison to human cases, have nonetheless proven valuable in deepening our understanding of CSNB2 pathology and the function of Cav1.4 in visual processing.

The naturally occurring mouse model known as nob-2 presents a phenotype that closely resembles human CSNB2 [86,102]. This similarity made nob-2 mice an initial tool for studying the underlying mechanisms of CSNB2. In nob-2 mice, the Cav1.4 protein can be observed in its full length. However, alternative splicing produces also a second mRNA variant, accounting for approximately 10% of the total mRNA population. This minor variant results in a subtle alteration at the N-terminus of the protein. Consequently, this modification leads to a substantial decrease in overall protein levels [102]. These alterations in the Cav1.4 protein mirror some of the genetic variations observed in human CSNB2 patients [10]. ERG studies on nob-2 mice have revealed detectable but reduced responses compared to wild-type mice. The b-wave and oscillatory potentials are reduced and the a-wave does not show any alterations [91]. The partial preservation of the b-wave under dark adapted conditions in these mice is similar to that seen in CSNB2 patients with pathogenic CACNA1F variants. This finding provided a quantifiable measure of the impact of reduced Cav1.4 protein on visual processing [10,86].

The pathogenic I745T Cav1.4 variant, first identified in a New Zealand family with CSNB2 [103], has become a focal point in CSNB2 research due to its unique characteristics and impact on visual function. This missense mutation, corresponding to the position I756T in mice (Cav1.4-IT, see above), causes a significant alteration in the calcium channel’s gating properties. In heterologous expression systems, the Cav1.4-IT variant induces a remarkable −30 mV shift in the voltage-dependence of channel activation [104]. This shift is likely attributed to increased channel conductance and higher open probability [105] and similar to pathogenic variants in the *CACNA1D* gene [106]. Mouse models carrying the equivalent mutation have proven invaluable in understanding the human CSNB2 phenotype [64,85,87,101]. These mice display many characteristics observed in human patients with the same mutation [64], which validates the mouse model as a highly valuable tool for investigating the CSNB2 phenotype. ERG recordings in these mice show rod- and cone-driven a-waves, but there is a decrease in scotopic and photopic b-waves which reflect an effect on ON and OFF bipolar cell function similar to human ERGs [64]. Notably, Cav1.4-IT mice also demonstrate progressive retinal degeneration, a finding that raises questions about potential long-term effects in human patients [87]. ERG recordings from the original New Zealand family did not initially suggest photoreceptor degeneration. However, the lack of long-term clinical investigations, such as regular ERG testing and optical coherence tomography (OCT), leaves this possibility open for further exploration. Interestingly, a separate study using spectral-domain OCT revealed differences in retinal layer thickness between CSNB2 patients and myopic controls, hinting at potential structural changes associated with the condition [99]. Further longitudinal studies in human patients are needed to determine if similar changes occur over time in affected individuals.

The pore-forming subunit Cav1.4α1 is, however, not the only protein in the channel complex implicated in retinal diseases. Its auxiliary subunit α2δ-4, which plays a crucial role in membrane trafficking (see also above). Several α2δ-4-KO mouse models have been developed, including those by Wang et al. [98] and a more recent model generated using gRNA targeting Exon 2 by Kerov et al. [101]. Additionally, Wycisk and colleagues [73] investigated a spontaneous frameshift mutation in mice (c.2367insC) that results in a truncation of the α2δ-4 protein, causing a reduction in the scotopic a- and b-wave. Although the authors were unable to record the cone ERG at any age, they characterized this pathogenic frameshift variant as causing cone-rod dystrophy. Notably, the architecture of cone synaptic ribbon structures appeared unaffected in the mouse model investigated by Wang et al. (2017). In contrast, Kerov and colleagues observed structural defects in α2δ-4-KO cones, where 3D reconstruction via EM revealed that most cone ribbons lacked a synaptic triad and that cone bipolar cells were sprouting, despite normally distributed ribbons. All the mouse models described exhibited no scotopic b-wave [73,98,101] and a significant reduction in the a-wave (only in Wycisk et al.), indicating rod dysfunction. This phenotype was further confirmed by a significantly longer time to detect the escape platform in a visually guided swim assay in both reports [98,101]. While ERG recordings suggested signal transmission in cones (Kerov et al., reported no photopic b-wave; Wang et al., noted a reduced photopic b-wave), and patch clamp recordings of cone bipolar cells corroborated these findings, visually guided swim assays under photopic conditions showed no significant differences in both mouse models. The diverse phenotypical characteristics observed in mice were similarly observed in human patients. Various pathogenic variants in patients have been investigated by different groups. Wycisk and colleagues [67] studied a truncation variant in exon 25 (c.2406C>A) in two siblings, which led to mildly reduced visual acuity, subnormal cone response, and photophobia. Another mutation causing a deletion of exons 17–26 led to a similar phenotype. Ba-Abbad and colleagues discovered other pathogenic variants in patients through whole-genome sequencing [93]. They identified a nonsense mutation in exon 19 (c.1882 C>T), likely subject to non-mediated mRNA decay, with patients exhibiting a distinctive multiphasic b-wave in single flash cone ERG and delayed, subnormal pattern ERG. Another patient with a homozygous deletion of exons 17–26, including a two-nucleotide insertion, showed the same photopic phenotype as those with the exon 19 mutation. However, at the follow-up they noted a possible rod dysfunction. Clinical findings in patients with mutations in the *CACNA2D4* gene revealed that they did not suffer from night blindness but rather from a mild form of cone dystrophy [93]. Only one patient showed a possible rod dysfunction.

Overall, a lack of α2δ-4 protein appears to impact the retinas of mice and humans differently. In mice, the phenotype seems more pronounced in the rods, as evidenced by the loss of the scotopic ERG b-wave. In contrast, humans exhibit a more distinct phenotype in the cones, except for one patient who shows a possible rod dysfunction.

### Lessons from Cav1.4-related CSNB2 models

The impairment of Cav1.4 function in both rod and cone photoreceptors triggers a cascade of adaptive responses in second-order neurons, including rod and cone bipolar cells as well as horizontal cells. One of the most notable adaptations is dendritic sprouting, where these second-order neurons extend their dendrites beyond their normal boundaries into the outer nuclear layer of the retina. There this sprouting results in the formation of ectopic synapses, which are synaptic connections formed in atypical locations within the retinal structure. These structural changes are, however, not limited to postsynaptic elements. Presynaptic components, including ribbon synapses and associated proteins, are also found mis-localized in the ONL. This ectopic placement of synaptic machinery is a hallmark of Cav1.4-related retinal disorders and has been consistently observed across various studies and mouse models [27,64,81,85–87,89,100,101,107,108]. In Cav1.4-KO mice, postsynaptic remodeling begins earlier in development, around postnatal day 11 (P11). In contrast, mice with the Cav1.4-IT mutation show a delayed onset of these changes [90]. The severity and timing of these synaptic alterations can, however, vary depending on the specific Cav1.4 mutation or knockout and likely reflects the varying degrees of presynaptic dysfunction caused by different Cav1.4 alterations.

Interestingly, while both rod and cone pathways are affected in CSNB2 mouse retinas, there may be differences in how these cell types respond to Cav1.4 dysfunction. For instance, in the Cav1.4-G369imouse model, cone synapses show a greater degree of preservation compared to rod synapses [27,28]. Detailed analyses have shown that presynaptic proteins and postsynaptic signaling complexes in cone synapses of Cav1.4-G369imice are enriched and structurally organized similarly to those in WT mice. However, these structures occupy a larger volume, suggesting an expansion of the cone pedicle with a lower intensity of synaptic proteins, indicating a spread rather than an increase in protein levels [28]. The structural complexity of cone synapses in Cav1.4-G369imice with some errors in postsynaptic wiring suggests an attempt to increase synaptic connectivity to compensate for the loss of Cav1.4 function. This contrasts with rod synapses, where the lack of Cav 1.4 Ca^2+^ signals lead to morphological abnormalities, such as shorter and fragmented ribbons, and a failure to develop mature synaptic structures. Functional ERG assessments in Cav1.4-G369imice indicated that the b-wave amplitude was reduced but still measurable and significantly larger than that observed in Cav1.4-KO mice at the highest light intensities [28]. Notably cone synaptic responses in Cav1.4-G369imice support vision-guided behavior under photopic (light) conditions, unlike rod synapses, which show significant deficits. This preservation allows Cav1.4-G369i mice to perform visual tasks effectively in light conditions, whereas they struggle under scotopic (dark) conditions, similar to Cav1.4-KO mice. The presence of Cav3 channels in Cav1.4-G369imice might compensate for the loss of Cav1.4, maintaining cone synaptic function and visual behavior under light conditions, but not in the dark.

According to the study by Knoflach et al., ERG recordings from Cav1.4-IT mice also showed severely reduced b-wave amplitudes under photopic conditions [64]. The study by Zanetti et al. revealed a more complex picture of synaptic reorganization in the retinal circuitry, and thereby another remarkable adaptability despite undergoing structural changes [89]. While their morphology was altered, cones maintained their crucial function of releasing glutamate, the primary neurotransmitter in the visual signaling pathway, in whole-cell patch-clamp recordings of postsynaptic horizontal cells as a read-out for transmitter release from cone presynaptic terminals. Thus, their findings suggested that the primary defect occurred not in the cone photoreceptors themselves, but in the postsynaptic cone bipolar cells. Functional data indicated a postsynaptic defect in cone bipolar cells, a finding further substantiated by the observation that most cone bipolar cells exhibited sprouting, while horizontal cells maintained their synaptic contacts with cones [89]. These findings highlight the differential responses of various retinal cell types to changes in cone photoreceptor morphology. While cone bipolar cells showed significant remodeling, possibly attempting to compensate for altered synaptic input, horizontal cells demonstrated resilience by preserving their functional connections with cones. This selective synaptic reorganization underscores the complexity of retinal circuitry and its capacity for adaptation. It also emphasizes the importance of studying not just individual cell types, but the entire network of synaptic connections in the retina to fully understand visual processing and potential therapeutic targets for retinal disorders. Together these findings collectively underscore the critical role of Cav1.4 in maintaining proper retinal synaptic architecture and function, while also revealing the retina’s capacity for structural adaptation in response to signaling deficits. The differential impact of Cav1.4 dysfunction on rod versus cone pathways might indeed contribute to the variability in visual symptoms observed in human patients with CSNB2 and related disorders. Gaining insight into these compensatory mechanisms may potentially guide the development of targeted treatments for visual impairment associated with Cav1.4 dysfunction. In ERGs of β2-KO mice, the b-wave amplitude was significantly reduced across all light intensities, while the a-wave remained unaffected. Interestingly, a low-amplitude positive component was detected in dark-adapted ERGs, suggesting that β2-KO mice retain some level of signal transmission in rods. Similarly, the b-wave amplitude was markedly reduced in cones [104,105]. As the β2 protein plays a vital role in targeting the pore-forming Cav1.4 protein to the plasma membrane, most of the observed phenotypes can be linked to the absence of Cav1.4 protein at the active zone. To date, no studies have investigated the potential effects of subretinalinjection of the β2 subunit on increasing channel localization at the synapse in mice. Given its smaller gene size, the β2 subunit could be more easily packaged into adeno-associated viruses (AAV). Enhancing β2 subunit levels might restore Cav1.4 channel protein in CSNB2 loss-of-function variants, where protein is reduced [10]. In cardiac cells, Télémaque and colleagues [109] demonstrated that even parts of the β2 subunit can at least modulate channel function. They reported two truncated β2 subunits containing either the BID and C-terminus or the BID alone, which bind intracellularly to Cav1.2 channels. However, these variants lack the necessary motifs to target the Cav1.2 channel complex to the cell surface and significantly reduce L-type Ca^2+^ current density in HL-1 cells. Still, this research exemplifies how we might use auxiliary subunits of the Cav channel complex to fine-tune or compensate for Cav1.4 CSNB2 variants, presenting a potential new therapeutic approach.

Research on mice lacking the α2δ-4 protein has revealed significant changes in the structure and function of photoreceptor synapses [91,92]. The disruption of presynaptic Ca2+ signaling and alterations in photoreceptor synapse structure observed in the mouse model suggests that similar mechanisms may underlie visual deficits in humans with α2δ-4 dysfunction. Specifically, the absence of α2δ-4 in mouse retinas leads to a gradual loss of presynaptic calcium channels in both rod and cone photoreceptors, resulting in compromised synaptic integrity. Similar to Cav1.4, α2δ-4 subunits play a crucial role in organizing synaptic ribbons and regulating the voltage sensitivity of Ca1.4 channels in rod photoreceptors [101]. Their absence leads to the abolishment of rod synapse formation and disrupts the clustering of postsynaptic mGluR6 receptors. In cone photoreceptors, while α2δ-4 is essential for Ca1.4 channel function, it is not required for ribbon organization or synaptic transmission [101].

The research team led by Martemyanovfocused on ELFN1, a protein that mediates a transsynaptic link between rod ribbon synapses and mGluR6 through α2δ-4 (Figure 1). Their study revealed that ELFN1 deficiency results in impaired scotopic transmission, while leaving photopic transmission intact. This selective effect implies that cone photoreceptors employ different mechanisms for synaptic transmission compared to rods [91]. In a more recent study by Cao et al. [110] another ELFN isoform, ELFN2, was identified, being exclusively localized in cone photoreceptors. Interestingly, knocking out ELFN2 did not disrupt transmission from cones to ON-cone bipolar cells. However, a double knockout of both ELFN1 and ELFN2 completely prevented synaptic transmission in cones, suggesting a compensatory relationship between these two molecules. Cao and colleagues further investigated the post-developmental plasticity of rod photoreceptors by selectively disconnecting and reconnecting them in mature mice after retinal circuit development was complete [110]. Using a tamoxifen-inducible Cre mouse model to knock out ELFN1 the researchers temporarily disabled ELFN1 in adult mice. Subsequently, they successfully restored synaptic function by reintroducing ELFN1 through rAAV gene therapy. Their findings revealed that adult rod photoreceptors could reintegrate both structurally and functionally into the existing retinal network. Most notably, the adult mice with newly reconnected rods acquired high-sensitivity vision, even if they lacked it since birth, demonstrating unexpected plasticity in the mature retinal circuitry organization beyond the critical developmental period. Although no CSNB2-causing variants in the ELFN1 gene have been identified to date, this research raises the possibility of restoring vision after development. α2δ-4 subunits play also several important roles in the organization of cone photoreceptor synapses. α2δ-4 is crucial for the function of voltage-gated Cav1.4 LTCCs in cone photoreceptors and for maintaining Cav1.4 channels in cone terminals, although the loss of these channels occurs later in cones compared to rods. While cone synapses are less affected than rod synapses, there are still notable changes in their organization. Although ribbons in cone pedicles of α2δ-4-KO mice appear mostly normal in structure less than one-third of cone ribbons show the expected triadic organization of processes at ribbon sites in the absence of α2δ-4. Despite these structural changes, cone synapses mice maintain some level of functionality, as evidenced by better visual performance under photopic (cone-mediated) conditions compared to scotopic (rod-mediated) (see above). The slower loss of Cav1.4 channels in cone terminals compared to rod terminals suggests the presence of compensatory mechanisms in cones, which may help preserve some cone-mediated vision in certain conditions.

These insights enhance our understanding of retinal pathologies associated with α2δ-4 dysfunction. Recognizing the progressive loss of Cav1.4 channels – initially in rod terminals and subsequently in cone terminals – can inform future research on potential therapeutic strategies. This knowledge may lead to the development of targeted therapies aimed at preserving or restoring Cav1.4 channel function, thereby potentially slowing or preventing vision loss in affected individuals.

### Current conceptual approaches for reversing Cav1.4 dysfunction and limitations

Laird et al. explored the potential for rescuing rod photoreceptor synapses in Cav1.4-KO mice by reintroducing the Cav1.4α1 subunit through in vivo electroporation [111]. Although only 10% of rods were transfected, the re-introduction of Cav1.4 rescued synaptic development, as evidenced by the appearance of PSD-95 and the presence of elongated synaptic ribbons. When *cacna1f* expression was induced in mature animals, similar restorative effects were observed, indicating that synaptic development could be rescued even in adult mice with established synaptic disease. In fully developed animals, the Cav1.4 protein was found to be spread out across the photoreceptor terminals, instead of being concentrated near the synaptic ribbon as expected. This dispersed arrangement implies that the organization of these proteins may not be ideal for optimal functioning. Despite this, approximately 25% of the treated animals successfully passed a vision-guided water maze test, indicating functional recovery. This suggests potential improvements in visual processing and demonstrates the robust nature of rod synaptic plasticity, even in adult animals with late-stage synaptic disease; despite the critical role of Cav1.4 in synaptogenesis.

Similarly, Waldner and colleagues explored a complementary strategy, employing Cre-driven, retina-specific *cacna1f* expression during prenatal development in a CSNB2 mouse model [112]. Their approach utilized Cre-driven, retina-specific expression of*cacna1f* during prenatal development via Pax6. Immunohistochemical analyses revealed that retinal sections with transgenic *cacna1f* expression exhibited wild type-like morphology, including restored photoreceptor and synaptic structures. Although the Despite mosaic transgene expression, immunohistochemical analyses revealed partial restoration in affected areas. Optokinetic response (OKR) testing suggested improvements in visual acuity, but statistical analysis of OKR and ERG data was limited due to small sample sizes. The study also assessed visual function through optokinetic response (OKR) analysis, which suggested some improvement in visual acuity in treated mice. However, due to small sample sizes, statistical analysis of ERG and OKR data was not possible. Both studies highlight the potential avenues for compensating the loss of endogenous Cav1.4 channels; however, they face significant limitations and challenges. Laird et al.’s electroporation strategy resulted in sparse transfection and statistically insignificant functional improvements, likely due to retinal detachment and unilateral treatment.

Formation of tamoxifen-inducible Cav1.4 channels via electroporation results in a sparse transfection causing only minor, statistically insignificant improvement in the functionality of the retina, as assessed by the visually guided water maze test

That’s likely why no changes were observed in the b-wave during ERG experiments. Laird et al. comment on the lack of functional improvement to a probable retinal detachment caused by in vivo electroporation. Moreover, they injected only one eye, which may also have contributed to the diminished performance in the water maze test (119).

On the other hand, Waldner et al.’s transgenic approach aims to improve the morphology like wild-type retinas, but suffered from mosaic expression and lacked in-depth functional analysis.

An in-depth analysis was not performed. Similar to Laird et al., they were only able to partially restore visual function, possibly due to the limited and mosaic-like expression pattern of the protein

These studies advance CSNB2 research by demonstrating that loss-of-function variants in the *cacna1f* gene could potentially be compensated, even in mature mouse retinas. However, their limitations highlight why these approaches are not yet viable for clinical application in humans: a) neither study provided evidence of a robust, widespread restoration of Cav1.4 function; b) consistent improvements in retinal signaling were not achieved, and c) issues such as inefficient transfection, incomplete protein localization, and limited scale of functional recovery were observed. These challenges underscore the pressing need for more effective and clinically translatable strategies. While gene-based approaches for CSNB2 show potential, significant hurdles must be overcome before they can become a realistic treatment option.

## Conclusion

This review illuminates the pivotal role of Cav1.4 LTCCs and how their complex interactions with auxiliary β and α2δ subunits significantly influence channel properties and function in photoreceptor synapses. Research on small animal models has significantly contributed to our understanding of retinal channelopathies, like CSNB2, caused by mutations in the corresponding genes. While these animal models have limitations in fully replicating human retinal physiology, they have nonetheless provided crucial insights into CSNB2 and revealed potential compensatory mechanisms that can occur even in fully developed retinas when there are loss-of-function variants in the *CACNA1F* gene. The review also emphasizes the potential of Cav1.4 channels as targets for gene therapy in retinal diseases. Future research in this field will have to increase sample sizes to improve statistical power and refine gene therapy approaches to enhance functional improvements. Still, the development of gene therapy for *CACNA1F*-related disorders encounters a significant challenge due to the size of the *CACNA1F* gene, which exceeds the packaging capacity of a single AAV vector; for review see [113]. To address this limitation, researchers must explore alternative strategies, such as utilizing dual AAV vector systems that can accommodate larger genetic payloads by splitting the gene across two vectors, or investigating other gene delivery methods capable of handling larger genetic sequences [9]. This size constraint also presents a crucial hurdle in translating *CACNA1F* gene therapies from laboratory research to clinical applications, highlighting the need for innovative approaches in vector design and delivery techniques. Those advancements will be crucial in translating basic research findings into effective therapies for retinal channelopathies and other visual impairments associated with Cav1.4 dysfunction.

## Data Availability

Data sharing is not applicable to this article as no new data were created or analyzed in this study.
